# Norovirus Protease Structure and Antivirals Development

**DOI:** 10.3390/v13102069

**Published:** 2021-10-14

**Authors:** Boyang Zhao, Liya Hu, Yongcheng Song, Ketki Patil, Sasirekha Ramani, Robert L. Atmar, Mary K. Estes, B. V. Venkataram Prasad

**Affiliations:** 1 Department of Molecular Virology and Microbiology, Baylor College of Medicine, Houston, TX 77030, USA; boyangz@bcm.edu (B.Z.); kpatil@bcm.edu (K.P.); ramani@bcm.edu (S.R.); robert.atmar@bcm.edu (R.L.A.); mestes@bcm.edu (M.K.E.); 2 Verna and Marrs McLean Department of Biochemistry and Molecular Biology, Baylor College of Medicine, Houston, TX 77030, USA; lhu@bcm.edu; 3 Department of Pharmacology and Chemical Biology, Baylor College of Medicine, Houston, TX 77030, USA; ysong@bcm.edu; 4 Department of Medicine, Baylor College of Medicine, Houston, TX 77030, USA

**Keywords:** norovirus, protease, structure, antivirals

## Abstract

Human norovirus (HuNoV) infection is a global health and economic burden. Currently, there are no licensed HuNoV vaccines or antiviral drugs available. The protease encoded by the HuNoV genome plays a critical role in virus replication by cleaving the polyprotein and is an excellent target for developing small-molecule inhibitors. The current strategy for developing HuNoV protease inhibitors is by targeting the enzyme’s active site and designing inhibitors that bind to the substrate-binding pockets located near the active site. However, subtle differential conformational flexibility in response to the different substrates in the polyprotein and structural differences in the active site and substrate-binding pockets across different genogroups, hamper the development of effective broad-spectrum inhibitors. A comparative analysis of the available HuNoV protease structures may provide valuable insight for identifying novel strategies for the design and development of such inhibitors. The goal of this review is to provide such analysis together with an overview of the current status of the design and development of HuNoV protease inhibitors.

## 1. Introduction

Human norovirus (HuNoV), a single-stranded, positive-sense RNA virus in the family *Caliciviridae*, is the leading cause of acute gastroenteritis around the world [1]. HuNoV diseases cause a significant economic burden, with an estimated USD 65 billion in costs globally each year because of direct health care costs and loss of productivity [2,3]. As the major cause of gastroenteritis outbreaks in industrialized nations [4], over 20 million symptomatic infections occur in the United States annually [5]. Even though the disease is self-limiting and generally lasts for 28–60 h, it still results in more than 70,000 hospitalizations and about 800 deaths annually in the United States [5]. HuNoV-associated acute gastroenteritis is characterized by vomiting, diarrhea, and abdominal pain, with nausea, fever, chills, and fatigue happening in a proportion of patients. However, chronic disease and severe complications can occur in immunocompromised patients. Chronic gastroenteritis can last for months or even as long as nine years in immunocompromised patients and can be life-threatening in patients with persistent dehydration, malnutrition, and dysfunction of the intestinal barrier [6,7]. Immunocompromised patients, especially those who are on immunosuppressive therapy, or before or after stem-cell transplant or solid organ transplant, spend considerable time in hospital and community settings where a number of surfaces may be contaminated with HuNoV [8] and serve as a source for transmission [9]. Infection may also be acquired in the community [10]. Therefore, immunocompromised patients are highly vulnerable to HuNoV gastroenteritis. The development of effective therapeutic options will be helpful for these patients.

Currently, there are no licensed HuNoV vaccines or antiviral drugs available, and the treatment of patients with HuNoV gastroenteritis is primarily supportive by preventing dehydration. Several HuNoV vaccine candidates are in different stages of development [11,12,13,14,15,16,17,18,19,20,21,22,23]. These vaccine candidates are developed based on virus-like particles (VLPs) and capsid proteins. Some vaccine candidates have shown promising results in inducing antibodies to correlate with protection against HuNoV gastroenteritis [12,17,18,19]. Antibodies blocking the binding of HuNoV VLPs to host glycans can be detected by different assays [11,12,24], and serum antibodies with this activity have correlated with serum neutralizing activity [25]. Several studies have demonstrated that intranasal or intramuscular administration of HuNoV vaccines that are designed based on VLPs can prevent HuNoV illness, but field efficacy trials are needed to fully understand the ability of vaccination to protect against illness caused by a variety of HuNoV strains [11,13,26].

Immunization with a given genotype vaccine or infection with the same genotype may not produce blocking antibody, a surrogate for neutralization, against other circulating genotypes, especially in young children and immunocompromised individuals [27,28]. As one of five recognized genera in the family *Caliciviridae*, the *Norovirus* genus is divided into 10 different genogroups (GI–GX) based on the amino acid sequence analysis of VP1 capsid protein [29]. Genogroups are further subdivided into genotypes based on amino acid sequences of the major capsid protein VP1 and nucleotide sequences of RNA-dependent RNA polymerase (RdRp) [30]. Since the first identification in fecal specimens by electron microscopy in 1972 [31], HuNoVs have caused many epidemics and endemic infections, and at least 35 human-infecting genotypes in GI, GII, GIV, GVIII, and GIX genogroups have been identified. Currently, GII.4 strains (genogroup two, genotype four) cause the majority of HuNoV infections worldwide, although other genotypes such as GII.2 and GII.17 occasionally have been predominant in certain geographic areas [32,33,34]. Since 1996, GII.4 variants have caused about 80% of global outbreaks [35], with six pandemics, including the US1995/96 strain in 1996 [36,37], the Farmington Hills strain in 2002 [38,39], the Hunter strain in 2004 [40], the Minerva strain in 2006 [41], the New Orleans strain in 2009 [42], and most recently, the Sydney strain in 2012 [43]. Therefore, the challenge is to develop broadly protective vaccines. Cross-genotype antibodies were elicited in healthy US adults by a GI.1/GII.4 bivalent VLP vaccine candidate and protection was seen against moderate/severe acute gastroenteritis caused by a heterotypic GII.2 virus [26]. How long protection lasts and whether broad heterotypic protection can be induced in other populations, especially in children, remains to be determined.

Furthermore, the evolution of HuNoV, through the accumulation of point mutations and recombination, especially in the capsid gene, may help HuNoV escape host immunity induced by vaccination [44]. The major driving force of GII.4 HuNoV evolution is a copy-choice model of intra-genotype recombination, which predominantly occurs in a conserved and overlapping region in the HuNoV genome that codes for RdRp and VP1 [45,46]. However, atypical recombination breakpoints located within the VP1 capsid sub-genome have been observed in 15 strains of the GII.2 genotype [47]. Several studies have suggested that these atypical recombination breakpoints may also be present within the VP1 capsid sub-genome of GII.4 genotypes [48,49,50], as they are present in three GII.4 variants [51]. Recombination within the major capsid protein VP1 has large potential to create antigenically novel GII.4 variants. In addition, novel epidemic subtypes can emerge within months after the acquisition of enough mutations in HuNoV strains given that the genetic drift rate of HuNoV is about 2–9 × 10^−3^ substitutions per nucleotide per year [52]. GII.4 strains even have a higher mutation rate in immunocompromised patients compared to in other patients [53]. Therefore, the development of a multivalent VLP-based vaccine that can elicit protection against HuNoV variants absent in the vaccine or periodic updating of the vaccine to combat the newly circulating HuNoV variants may be necessary. Whether evolution will shorten the duration of protection and reduce the cost-effectiveness of HuNoV vaccines remains to be determined [54]. Moreover, the duration of vaccine protection is still debated, considering that early studies suggested protection after natural HuNoV infection is not long-lasting, although more recent modeling studies suggest immunity may last more than eight years [55]. Immunocompromised patients without a functional humoral immune response may not be protected by vaccination. Therefore, these groups at high risk of death and severe complications could benefit from immediate and robust post-infection therapeutic intervention strategies, including small-molecule drugs that inhibit HuNoV replication.

## 2. Structural Features of HuNoV Protease

Viral proteins encoded by the HuNoV genome, especially the ones that mediate HuNoV replication, are potential targets for developing small-molecule drugs. The RNA genome of HuNoV, approximately 7.7 kb long, is divided into three open reading frames (ORFs). The 5′ proximal ORF1 encodes a large polyprotein that is cleaved into six non-structural proteins by a viral protease [56]. ORF2 encodes a major capsid protein VP1, and ORF3 encodes a minor capsid protein VP2. VP1 capsid contains a conserved shell (S) domain and a protruding (P) domain that is subdivided into P1 and P2 subdomains. The surface P2 domain interacts with the histo-blood group antigens (HBGAs) present on the surface of the epithelial cell to mediate initial viral attachment followed by virus entry into cells [57,58,59]. The released VPg-linked viral RNA genome is then processed by the cellular translation machinery via VPg-mediated translation, leading to the synthesis of a polyprotein, the capsid protein VP1, and the minor capsid protein VP2 [60]. The non-structural proteins released by the proteolytic cleavage of the polyprotein form a replication complex in the cytoplasm, followed by genome replication. Utilizing both de novo and VPg-dependent initiation mechanisms, the RdRp generates the genomic and the subgenomic viral RNAs [61]. The replicated genomic RNA is then packaged into the virion. A viral protein that is structurally and functionally conserved should be considered as a target for the development of antiviral drugs. Although each stage of the HuNoV replication cycle can be a unique target for the development of antivirals, the viral protease is an appealing drug target because of its central role in the polyprotein processing essential for viral replication.

HuNoV protease cleaves the polyprotein encoded by the ORF1 at five sites to release six non-structural proteins with distinct functions, including the formation of membrane-associated replication complex (p48 [NS1/2], p41 [NS3], p22 [NS4]), initiation of genome translation (VPg [NS5]), cleavage of the polyprotein (Pro [NS6]), and replication of the viral genome (RdRp [NS7]), which are essential for the viral replication to proceed. Therefore, inhibition of the proteolytic activity of HuNoV protease would effectively hamper viral replication by abolishing the release of the non-structural proteins. The protease inhibitors described in the literature are mostly peptidomimetics that target particularly the GI.1 protease active site and block the protease substrate-binding pockets. Although these inhibitors show strong inhibition in the Norwalk virus (GI.1) replicon system at low micromolar concentrations, they show significantly reduced inhibition activity to GII.4 protease. As GII.4 variants have become the predominant genotype, causing 80% of global infections, it is important to develop protease inhibitors specific to GII.4 variants. Recent elucidation of protease structures of two GII.4 variants [62,63] indicate significant structural differences between the active sites and substrate-binding pockets of GI.1 and GII.4 proteases. The strategy of optimizing existing GI.1 inhibitors to combat GII.4 may not generate inhibitors with potent inhibition activity. Therefore, the identification of a novel class of small-molecule protease inhibitors that can effectively inhibit HuNoV across the genogroups should be a priority.

### 2.1. Overall Structure of HuNoV Protease—Similarities and Differences

**Figure 1:**
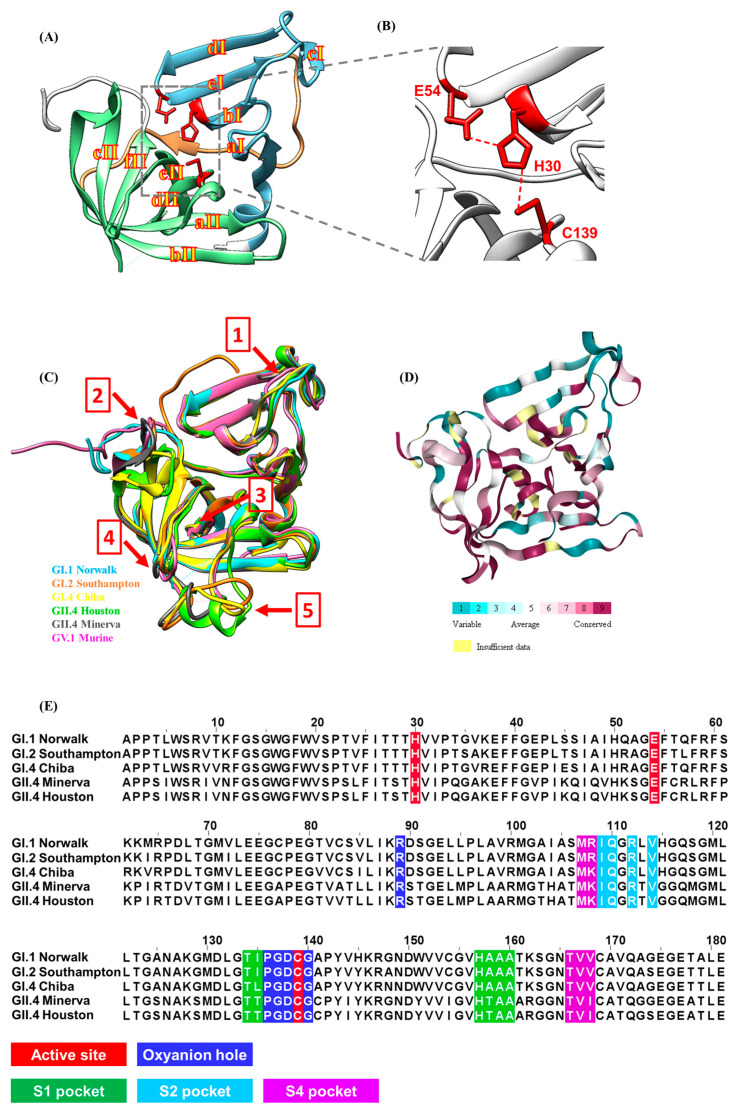
Three-dimensional structure of HuNoV protease and its active site. (**A**) Ribbon representation of Norwalk protease structure (PDB ID: 2FYQ). Domain I (cyan) is connected to Domain II (lime) via a large loop (orange). The catalytic residues are shown as red stick models. The β-strands are shown as yellow characters with red outline. (**B**) The active site of HuNoV protease contains three residues colored red. (**C**) Structures of all the published protease are overall conserved with several regions exhibiting conformation flexibility, indicated by the red arrows. The five regions are: (1) the flexible surface loop from aa 33 to 36, (2) the hairpin loop from aa 107 to 113, (3) the short loop from aa 147 to 150, (4) the β-hairpin loop from aa 162 to 164, and (5) the loop from aa 122 to 134 (**D**) Conservation of amino acid positions in HuNoV protease structures. The figure was generated with the ConSurf server [69]. (**E**) The amino acid residues are colored in red for the active site, blue for the oxyanion hole, green for the S1 pocket, cyan for the S2 pocket, and magenta for the S4 pocket. The GenBank accession ID for each sequence is Norwalk, NP_786949.1; Southampton, AAA92983.1; Chiba, AB042808.1; Minerva, EF684915; Houston, ABY27559.

Structural features of HuNoV protease provide important information for the design and optimization of a compound with the goal of developing a drug for clinical use. The atomic structures of proteases from several HuNoVs belonging to different genogroups and genotypes have been determined [62,63,64,65,66,67]. A HuNoV protease, typically around 181 amino acids in length, consists of an N-terminal domain I and a C-terminal domain II connected via a large loop (Figure 1A). HuNoV protease is classified as viral chymotrypsin-like cysteine protease, as it contains a reactive cysteine residue in its active site, which is located at the cleft between the two domains (Figure 1B). Domain I is formed from a two-turn α-helix followed by five β-strands (aI, bI, cI, dI, eI). The five β-strands are connected via loops to form a twisted antiparallel β-sheet. Domain II consists of six β-strands (aII, bII, cII, dII, eII, fII) joined by intervening loops that form an antiparallel β-barrel [64]. Structural alignment of all the six published HuNoV protease structures suggests an overall conserved three-dimensional structure conformation. Five regions, (1) the flexible surface loop from aa 33 to 36, (2) the hairpin loop from aa 107 to 113, (3) the short loop from aa 147 to 150, (4) the β-hairpin loop from aa 162 to 164, and (5) the loop from aa 122 to 134 (Figure 1C), however, do exhibit noticeable conformation variations and the conformational flexibility could be due to the exposure to the solvent. A ConSurf analysis of HuNoV protease showed that these regions are less conserved compared to the active site (Figure 1D). One structural feature of note is that the integrity of the active site is maintained by several hydrogen bonds. The hydrogen bonds formed between the sidechain nitrogen of Lys88 and the backbone carbonyl oxygen of Val9 and between the sidechain nitrogen of Arg8 and the backbone carbonyl oxygen of Thr69 are vital for protease activity [65]. These residues are highly conserved across HuNoV proteases and mutation of these residues abolishes the protease activity [68]. The overall conserved structural features, including the active site, the substrate-binding pockets, and the oxyanion hole, are the target regions for inhibitor design (Figure 1E).

### 2.2. Active Site of HuNoV Protease

The major proteolytic activity of HuNoV protease, which is to cleave the polyprotein to release the non-structural proteins essential for viral replication, takes place in its active site. The active site of HuNoV protease, which is located at the cleft between the two domains, consists of three residues responsible for proteolytic activity (Figure 1B). Cys139, located in the loop that connects strands cII and dII, is the nucleophilic residue that cleaves the target peptide bond. His30, located in the short α-helix that follows strand cI, functions as a base to deprotonate and polarize the Cys139 residue. Glu54 orients the imidazole ring of His30 to improve catalytic efficiency [64].

**Figure 2:**
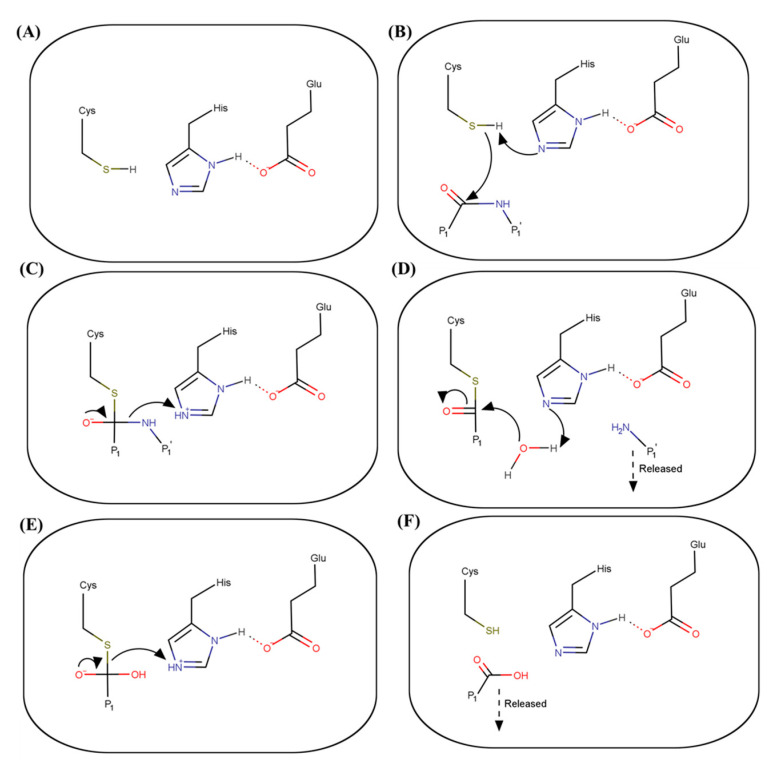
Proteolysis mechanism of HuNoV protease. (**A**) The three residues within the catalytic triad are at the rest state. (**B**) Binding of a substrate induces a conformational change, which leads to the removal of a proton on the thiol side chain of the Cys by the His ring. The active thiol then launches a nucleophilic attack on the carbonyl carbon of the substrate. (**C**) A covalent bond is formed between the substrate and the sulfur and a protease–substrate tetrahedral intermediate is formed. The His ring then donates a proton to the amide nitrogen of the substrate. (**D**) This leads to the breakage of the peptide bond, which releases one side of the substrate out of the active site, as indicated by the dashed arrow. The His ring then activates a water molecule, which launches a nucleophilic attack on the acyl-enzyme complex. (**E**) Attacking from the water molecule forms the second tetrahedral intermediate. The His ring again donates a proton to the intermediate complex. (**F**) The tetrahedral intermediate complex then decomposes. The substrate is released out of the active site. The enzyme is back to the native state. Red indicates an oxygen atom. Blue indicates a nitrogen atom. Green indicates a sulfur atom. Arrow indicates a transfer of an electron. Dashed arrow indicates the releasing of functional group from the active site.

Drugs targeting the active site are usually analogs that resemble the tetrahedral intermediate formed during the proteolysis process. The mechanism of proteolysis of chymotrypsin-like cysteine proteases is similar to that of serine proteases (Figure 2). The binding of a substrate induces changes in the positioning of three amino acids in the catalytic triad, which results in the activation of the thiol from the Cys and the formation of the protease–substrate intermediate complex. These changes include the shift of the negatively charged Glu towards the electron-rich His ring, which favors the removal of a proton from the thiol group on the sidechain of the Cys by the His, resulting in the activation of the thiol. The active thiol then launches a nucleophilic attack on the carbonyl carbon of the substrate peptide, resulting in the formation of a covalent bond between the substrate and the sulfur of the Cys to generate a tetrahedral intermediate. The oxyanion hole formed next to the active site stabilizes the negatively charged oxyanion of the tetrahedral intermediate through its positive charge. The transfer of the proton from the His to the amide nitrogen of the substrate then results in the breakage of the peptide bond, releasing one side of the substrate peptide out of the active site, and the formation of an acyl-enzyme complex. The covalent bond formed between the enzyme and the substrate needs to be broken so that the enzyme can return to its original state. A nucleophilic attack by a water molecule, activated by the His, results in a formation of a second tetrahedral intermediate. The second tetrahedral intermediate is then destabilized to release the carboxylic acid product, bringing the enzyme back to its original state. The three residues are highly conserved across HuNoV protease. Mutation of any of the three residues either compromises or completely abolishes the proteolytic activity [68].

**Figure 3:**
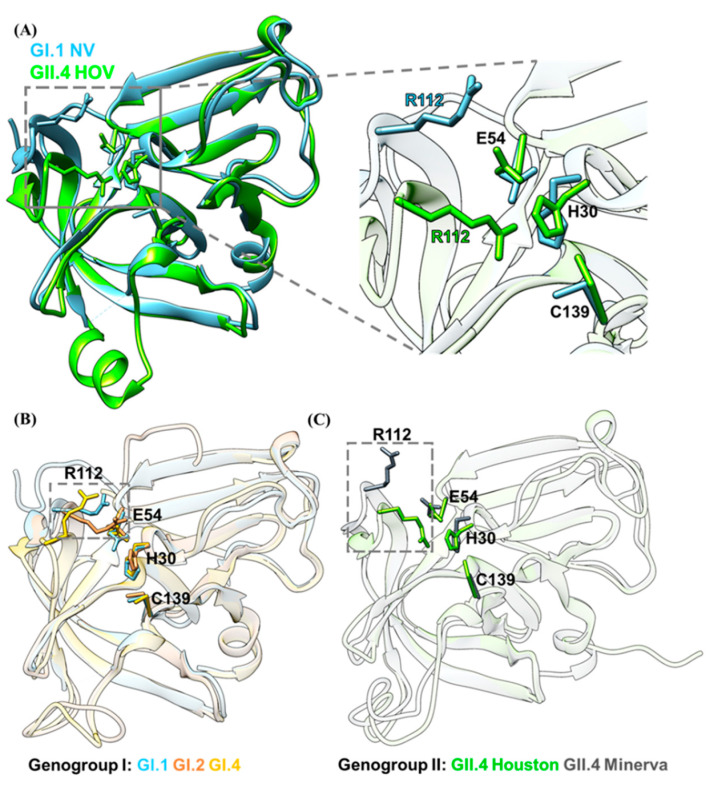
Interactions of the active site residues are affected by the Arg112 residue. (**A**) Comparison of the active sites of GI.1 Norwalk (cyan) and GII.4 Houston (green) depicts a different orientation of the His30 residue. In GI.1 Norwalk protease, the Arg112 residue is positioned away from the active site, therefore allowing His30 to interact with Glu54. However, in GII.4 Houston protease, such interaction between His30 and Glu54 is not observed, as the Arg112 residue is pointed toward the active site and interacts with His30. (**B**) Active site comparison within the GI genogroup protease (Norwalk, cyan; Chiba, yellow; Southampton, orange) indicates that the Arg112 residue (within the grey box) of the GI.2 Southampton protease is also pointed toward the active site. (**C**) Active site comparison within the GII genogroup protease (Houston, green; Minerva, grey) indicates that even though Houston and Minerva are in the same genogroup, the orientation of the active site residues are different due to the position of the Arg112 residue (within the grey box).

Although the overall structure and the composition of the active site are well conserved, there are subtle differences between the HuNoV proteases (Figure 3). In genogroup I, the interaction between His30 and Glu54 is referred to as a *syn* configuration in GI.1 Norwalk virus [64], whereas it is referred to as an *anti* configuration in GI.4 Chiba protease [65] (Figure 3B). However, such an interaction between His30 and Glu54 was not observed in GII.4 Houston protease [62]. Instead, His30 interacts with the sidechain of Arg112, a residue in the S2 substrate-binding pocket, via a cation-π interaction. This is because the Arg112 in GII.4 Houston protease is positioned toward the active site, causing a steric clash with the Glu54. The Glu54, therefore, has to orient away from the His30 to accommodate the Arg112 sidechain, which results in a completely different orientation of the imidazole ring of the His30 (Figure 3A). Close examination of the published protease structures indicates that both GII.4 Houston and GI.2 Southampton possess this Arg112 conformation variation, whereas the other GII.4 variant Minerva possess Arg112 conformation similar to GI.1 Norwalk and GI.1 Chiba. (Figure 3B,C). The orientation of the His30 imidazole ring in GII.4 Houston protease results in an unfavorable distance between His30 and Cys139. As a result, the deprotonation of the sulfhydryl group of Cys139 is impacted and the catalytic efficiency is influenced as the GII.4 Houston protease exhibits minimal activity at low pH. This subtle structural difference between the active sites of different genotypes hampers the development of broad-spectrum inhibitors.

### 2.3. Cleavage Sites and Substrate-Binding Pockets of HuNoV Protease

**Figure 4:**
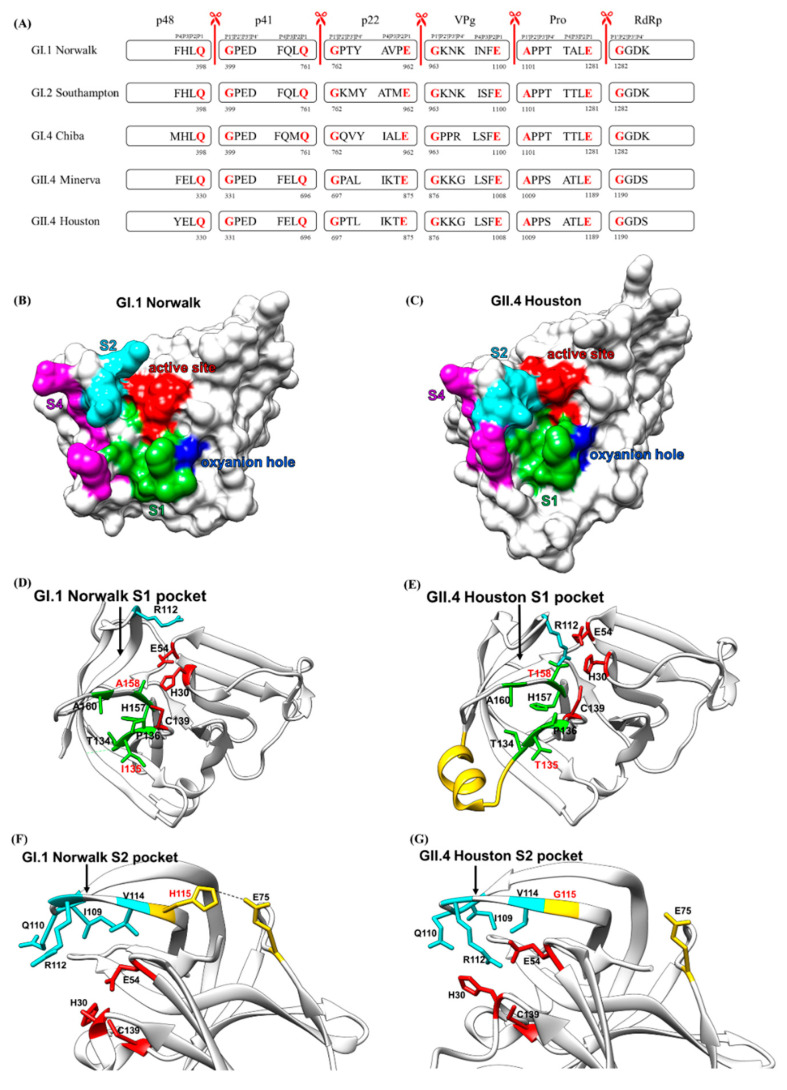
Protease cleavage sites and the substrate-binding pockets. (**A**) Cleavage sites of five HuNoV proteases. Each box indicating a non-structural protein contains the P4–P1 residues in its right side and the P1′–P4′ residues in its left side. P1 and P1′ residues are colored in red. (**B**) Surface representation of the substrate-binding pockets of Norwalk virus and (**C**) of Houston virus. (**D**–**G**) Structural comparison of Norwalk (PDB ID: 2FYQ) and HOV (PDB ID: 6NIR) proteases. (**D**,**E**) The residues mediating the binding specificity of the S1 pocket and the active site residues are colored in green and red, respectively. The structural differences between Norwalk and HOV are colored in cyan and yellow. (**F**,**G**) The residues mediating the binding specificity of the S2 pocket and the active site residues are colored in cyan and red, respectively. The structural differences of these proteases are colored in yellow.

For the active site to cleave the substrate at the right position, the substrate has to bind the substrate-binding pockets near the active site to be correctly aligned with the catalytic triad. The HuNoV protease cleaves the polyprotein at five cleavage sites, also known as scissile bonds. The residues surrounding the cleavage sites mediate the binding of the substrate to the HuNoV protease. The four N-terminal residues from the cleavage site are termed as P1, P2, P3, and P4 residues, and the four residues in the C-terminal of the cleavage site are termed as P1′, P2′, P3, and P4′ [70] (Figure 4A). The cleavage sites of calicivirus were first identified when the proteases of rabbit hemorrhagic disease virus (RHDV) [71] and feline calicivirus (FCV) [72] were found to preferentially cleave sites containing the P1 Glu residue with varied cleavage efficiency. The P1 cleavage sites of HuNoV protease were identified in the GI.2 Southampton virus when the 200 kDa polyprotein was cleaved cotranslationally into three major translation products at molecular masses of 113, 48, and 41 kDa, as revealed by a time-course in vitro transcription–translation experiment [73]. This led to the identification of two conserved P1 Gln-Gly cleavage sites found in the Southampton virus at amino acid positions 398 and 761. Site-directed mutagenesis of the Gln residues of the two Gln-Gly cleavage sites to Pro residues completely abolished the proteolytic activity [73]. Two additional P1 cleavage sites containing the Glu-Gly or Glu-Ala dipeptide were then identified in the Southampton virus when the cleavage products were expressed in the bacterial host [74]. Expression of the ORF1 cDNA of the genogroup 2 Camberwell virus in a eukaryotic system further confirmed the proteolytic processing at Glu-Gly and Glu-Ala in HuNoVs [75]. The protease processing map was then identified in the Norwalk virus through a mutagenesis study that indicated that P residues surrounding the P1 position also played vital roles in protease cleavage [76,77]. Norwalk virus protease cleaves at five different sites, Gln398Gly, Gln761Gly, Glu962Gly, Glu1100Ala, and Glu1281Gly, to release six non-structural proteins. These five P1 cleavage sites are well conserved in GII.4 Houston HuNoV even though the P2–P4 residues are different (Figure 4A).

#### 2.3.1. Substrate Recognition by the HuNoV Protease

**Figure 5:**
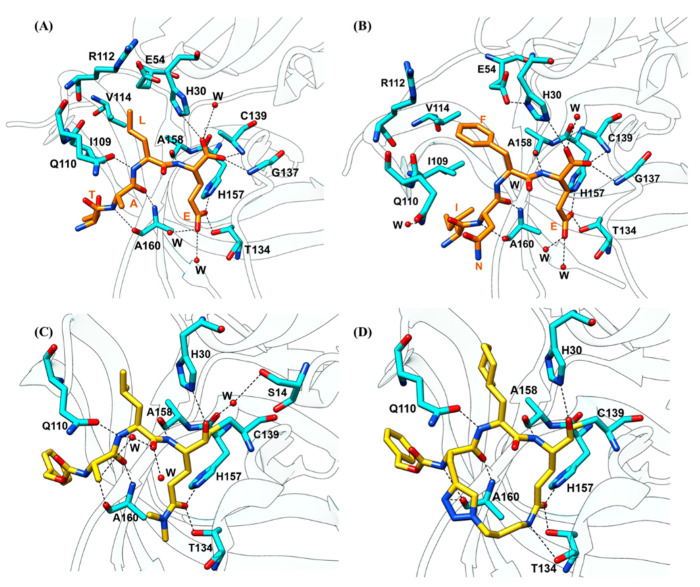
Binding and recognition of substrates and inhibitors to the HuNoV protease. (**A**) Binding of the TALE substrate (orange) to the Norwalk protease (cyan) (PDB ID: 4IN2). Water molecules are represented as red spheres. (**B**) Binding of the INFE substrate (orange) to the Norwalk protease (cyan) (PDB ID: 4IN1). (**C**) Binding of the Syc59 inhibitor (yellow) to the Norwalk protease (cyan) (PDB ID: 4INH). (**D**) Binding of the macrocyclic inhibitor 1 (yellow) to the Norwalk protease (cyan) (PDB ID: 6BIB). Oxygen and nitrogen atoms are labeled in red and blue, respectively.

The substrate recognition and specificity of HuNoV protease were characterized by structural studies. Typically, proteases have separate sites (S1–S4) that preferentially recognize the P1 to P4 residues (Figure 4B,C). The first protease–substrate complex structure was determined using genogroup I Chiba virus and substrate H-Glu-Ala-Leu-Phe-Gln-pNA [65]. Later, crystallization of GI.1 Norwalk HuNoV protease depicted the binding of TALE substrate (Figure 5A), corresponding to the Pro-RdRp cleavage site, and INFE substrate (Figure 5B), corresponding to the VPg-Pro cleavage site, to the active site [78]. The interactions between the TALE or INFE substrates and the protease are mainly located in the S1 and S2 substrate-binding pockets. Determination of the GII.4 Houston HuNoV protease structure then reveals the conformation variations of the substrate-binding pockets across different genogroups. The P1 Glu sidechain of TALE or INFE substrate fits tightly inside the S1 substrate-binding pocket, which is composed of hydrophobic residues in domain II, His157, and Thr134 in the cII-dII loop, and Ala160 of the eII β-strand, through a network of hydrogen bonds. The entrance of the S1 pocket is formed by residues Leu135, Pro136, and Ala160. The Nε2 of the imidazole ring of His157 and the hydroxyl group of Thr134 form hydrogen bonds with the Oε1 of the P1 Glu sidechain, whereas two water molecules form hydrogen bonds with the Oε2 of the P1 Glu sidechain. The sidechain of Tyr143 forms a hydrogen bond with the imidazole ring of His157 to properly orient His157 for substrate recognition [64]. Additional S1 pocket residues, including Ile135, Pro136, Ala158, and Ala160, also form hydrogen bonds with the P1 residue to properly position the terminal carbonyl carbon of the substrate for Cys139 cleaving (Figure 4D). These hydrogen bonds are vital for substrate recognition in the S1 pockets, as the mutations of the S1 pocket residues, which are conserved across Chiba HuNoV and Norwalk HuNoV proteases, completely abolish the protease activity [68].

The S1 substrate-binding pocket in GII.4 Houston protease is also composed of the hydrophobic residues, except for two residue replacements at position 135 from Ile to Thr and position 158 from Ala to Thr [62]. A major structural difference is that residues 122 to 134, which form the floor of the S1 pocket, form an α-helix in GII.4 Houston protease, whereas this stretch is unstructured in GI.1 Norwalk (Figure 4E). The bulky sidechain of the P2 residue is accommodated by the hydrophobic S2 substrate-binding pocket located in the bII–cII loop, which is composed of residue Ile109, Gln110, Arg112, and Val114. Ile109 is involved in the binding of both TALE and INFE substrates by providing a stabilizing van der Waal contact (Figure 4F). In the binding of the TALE substrate, the backbone amide of the P2 Leu forms a hydrogen bond with the Gln110 sidechain to stabilize the P2 recognition. In the binding of the INFE substrate, the bulkier P2 Phe sidechain makes hydrophobic interactions with Val114. Instead of forming a hydrogen bond with the Phe residue at P2, Gln110 is oriented away from the P2 residue and interacts with a water molecule. The S2 pocket of GII.4 Houston protease is considerably smaller compared to that of GI.1 Norwalk protease due to H115G mutation, which leads to a conformational change in the bII–cII loop (Figure 4G). The bII–cII loop in GI.1 Norwalk protease is stabilized by the hydrogen bonds between His115 and Glu75. Mutation of His115 to Gly115 in GII.4 Houston protease disrupts the hydrogen bonding, leading to the higher flexibility of the bII–cII loop. Therefore, residues in the bII–cII loop move closer to the active site to form a smaller S2 pocket. The S3 substrate-binding pocket is not well defined, as the P3 residues are highly variable. Hence, the P3 recognition interactions vary with the P3 chemistry, except for the conserved backbone-to-backbone hydrogen bond interactions involving the P3 residue and the Ala160 residue. The S4 substrate-binding pocket of Norwalk protease is formed by residues Met107, Arg108, Ile109, Thr166, and Val168. These residues are partially conserved in GII.4 protease.

#### 2.3.2. Substrate-Induced Conformational Changes

**Figure 6:**
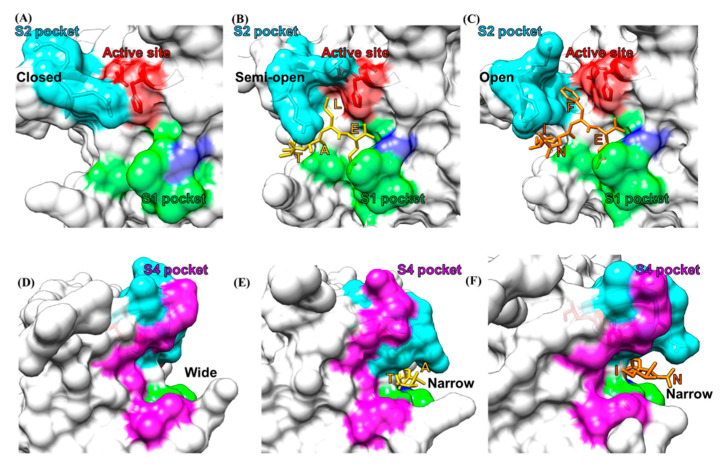
Conformational changes of the substrate-binding pockets induced by the binding of two substrates. (**A**) In the native unbound protease, the S2 pocket colored in cyan is in the closed conformation. The active site is colored in red and the oxyanion hole is colored in blue. (**B**) Binding of a TALE substrate (yellow) opens up the S2 pocket (PDB ID: 4IN2). (**C**) Binding of an INFE substrate (orange) further opens the S2 pocket (PDB ID: 4IN1). (**D**) The S4 pocket colored in magenta is widely open as the S2 pocket is closed. (**E**) The S4 pocket narrows when the TALE substrate (yellow) binds. (**F**) The S4 pocket becomes narrower when the INFE substrate (orange) binds.

The binding of the TALE or INFE substrates not only generates bonds that are important for substrate recognition but also induce conformational changes in the substrate-binding pockets (Figure 6). In comparison with the unbound protease structure, the S2 pocket exhibits noticeable conformational changes to accommodate variations in the P2 position of the TALE and INFE substrates. Without the binding of a substrate, the S2 pocket rests in its closed conformation as the bII–cII loop with the Gln110 residue pointing toward the active site (Figure 6A). The binding of the TALE substrate opens up the S2 pocket slightly to accommodate the Leu sidechain (Figure 6B), whereas binding of the INFE substrate further opens the S2 pocket to accommodate the bulkier Phe sidechain (Figure 6C). The semi-open and open states of the S2 pocket, which are mainly caused by the flexibility of the bII-cII loop that forms the major portion of the pocket, indicates that the protease has the ability to tolerate some level of conformational changes of the substrate by inducing structural changes in the substrate-binding pockets. This is also indicated by the correlated changes with the alterations in the S2 pocket exhibited by the S4 pocket upon substrate binding. The S4 pocket constricts when the S2 pocket is widened and opens up when the S2 pocket is constricted, since these two pockets share the bII–cII loop (Figure 6D–F). Therefore, the coordinated movement of the two pockets suggests that the substrate recognition and binding affinity could be determined by the P2 and P4 residues synergistically. Noticeably, the S2 pocket of the GII.4 Houston protease is even smaller than the closed state of the S2 pocket of the GI.1 Norwalk protease. The overall conserved sequence and conformation of these substrate-binding pockets provide an important target for developing the currently reported protease inhibitors.

### 2.4. Oxyanion Hole of HuNoV Protease

**Figure 7:**
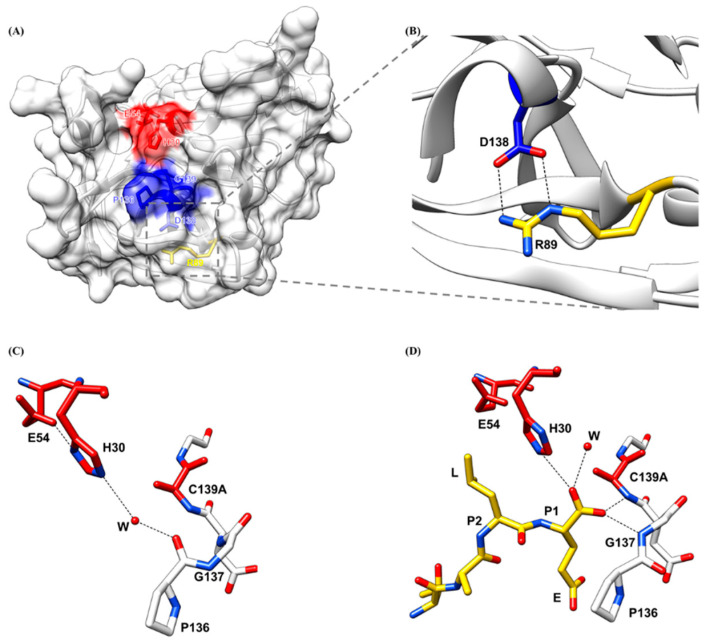
Oxyanion hole of the HuNoV protease. (**A**) The oxyanion hole colored in blue is next to the active site colored in red. (**B**) The amino acid residues involved in the hydrogen bonding to stabilize the oxyanion hole are colored in deep blue (Asp138) and yellow (Arg89). (**C**) In the native unbound protease, the active site colored in red interacts with the oxyanion hole colored in white through a water molecule (red sphere). (**D**) In the protease bound with a TALE substrate (yellow), the substrate interacts with the oxyanion hole colored in white though two hydrogen bonds. Oxygen and nitrogen atoms are colored in red and blue, respectively.

The binding of the substrate to the substrate-binding pockets and the cleavage of the substrate by the active site have to be stabilized by the viral oxyanion hole. An oxyanion hole is a pocket next to the active site of the viral protease that stabilizes the negative charge on the deprotonated oxygen of the tetrahedral intermediate formed during the transition state. The oxyanion hole is typically formed by the backbone amides or positively charged residues to promote proteolysis by protecting the negatively charged oxygen of the substrate from water molecules [79]. The oxyanion hole of HuNoV protease consisting of highly conserved sequence Pro136-Gly137-Asp138-Cys139-Gly140 (Figure 7A) is formed during the binding of the substrate P1 residue. A conformational change induced by the binding of the substrate is also observed in the oxyanion hole. In the unbound native protease structure, the backbone amide of Gly137 is positioned away from the active site and faces the solvent while the backbone carbonyl oxygen of Pro136 is pointed toward the active site and forms a hydrogen bond with the catalytic residue His30 via a water molecule (Figure 7C). When a substrate binds to the protease, the oxyanion hole is large enough to accommodate the P1 backbone and the terminal carboxyl group of the P1 residue interacts with the oxyanion hole residues. The positioning of the P1 carbonyl oxygens in the oxyanion hole induces the conformational change of the oxyanion hole, which triggers a flipping of the Pro136-Gly137 peptide, with the backbone carbonyl oxygen of Pro136 now positioning away from the active site, while the backbone amide residues of Gly137 face the active site to form a hydrogen bond with the substrate carbonyl oxygen. The interactions between the P1 carboxyl group and the oxyanion hole, with one P1 carbonyl oxygen forming a hydrogen bond with the backbone amide of Gly137 and the other P1 oxygen forming a hydrogen bond with the amide of Cys139, stabilize the binding of the substrate to the active site (Figure 7D). This conformational change induced by binding of the substrate to the active site is also observed in the Chiba HuNoV protease, where the binding of a tartrate substrate potentially causes the main chain of Pro136 to rotate away from the active site by almost 180 degrees, while the backbone amide of Gly137 turns inward [65]. Additionally, the oxyanion hole is stabilized by the hydrogen bond formed between the oxygen in the sidechain of Asp138 and the nitrogen in the sidechain of Arg89 (Figure 7B). Both Asp138 and Arg89 are conserved across HuNoV protease as proteases with Arg89Lys and Asp138Asn mutations are inactive [68]. Therefore, the oxyanion hole is a suitable drug target that needs to be considered in the current development of broad-spectrum inhibitors.

## 3. Development of Current HuNoV Protease Inhibitors

**Figure 8:**
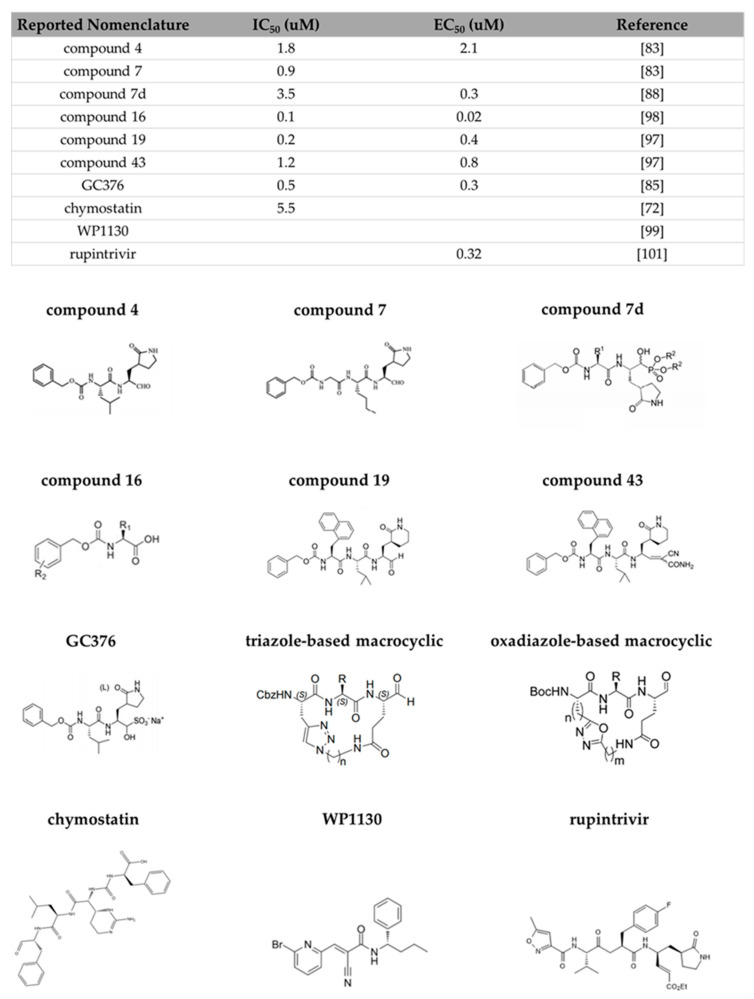
Inhibition potency of representative Norwalk protease inhibitors. The chemical structures and inhibition potencies of representative linear aldehyde peptidomimetic inhibitors (compound **4** and **7**), linear hydroxyphosphonate peptidomimetic inhibitor (compound **7**d), linear cyclohexylalanine peptidomimetic inhibitor (compound **16**), linear lactam peptidomimetic inhibitor (compound **19**), linear cyanoacrylamide peptidomimetic inhibitor (compound **43**), prodrug GC376, triazole-based and oxadiazole-based macrocyclic inhibitors, and commercially available inhibitors (chymostatin, WP1130, rupintrivir).

The development of functional antivirals was initiated long before the detailed three-dimensional structures of the viral protease structure became available by targeting the sequences of substrate cleavage sites of the viral protease. The fundamental strategy for designing an inhibitor for a cysteine viral protease is the synthesis of a peptidomimetic inhibitor. An oligopeptide consists of two or three P residues mimicking the substrate cleavage site with a reactive warhead linked to P1 residue, which will form a covalent bond with the active Cys residue to abolish the protease enzymatic activity. This strategy has been proven to be successful in blocking the rhinovirus cysteine viral protease in vivo when the inhibitor is linked with a C-terminal Michael acceptor group [80]. The first reported HuNoV protease inhibitor complex structure [66] denoted the interaction between the Southampton protease and a substrate-based inhibitor mimicking the EFQLQ sequence of the protease cleavage site linked with a C-terminal Michael acceptor group. The interactions are primarily via a hydrogen-bonding network in the form of an antiparallel β-sheet in the substrate-binding pocket. The design of a linear di-peptide or a tri-peptide peptidomimetic inhibitor is then adopted as a structural backbone for in-lab-synthesized HuNoV protease inhibitors that could be further transformed into molecules possessing important pharmacodynamic and pharmacokinetic properties by primarily modifying the chemical warhead that interacts with the HuNoV protease. The current synthesized HuNoV protease inhibitors reported in the literature can be classified into (1) linear peptidomimetic inhibitors, (2) prodrugs, and (3) macrocyclic inhibitors based on the design rationale (Figure 8).

### 3.1. Linear Peptidomimetic Inhibitors

The first series of 10 peptidomimetic inhibitors specifically targeting Norwalk protease were synthesized by adding an aldehyde warhead to a Gln surrogate [81]. From the synthesis pathway, the researchers found that the precursor alcohol warheads were essentially inactive, and only when they were transformed into an aldehyde warhead did the inhibitors exhibit inhibition activity. The N-terminal cap also mediated the activity of the inhibitors and is noted as a site for optimization in the later literature. One of the 10 inhibitors with less than 2 µM IC_50_ value also exhibited HuNoV replication inhibition with ~2 µM ED_50_ when it was tested in a cell-based replicon system (compound **4**). The compound formed hydrogen bonds with residues Thr134, Ala158, Gln110, and Ala160 of the protease. Replacement of the P2 Leu residue with a bulky hydrophobic residue such as Nle found in compound **7** greatly improved the inhibition potency as this substitution enhanced the fit of the P2 sidechain in the S2 pocket of the protease.

Structural analysis of the peptidomimetic linear inhibitors revealed that several protease residues are essential for mediating ligand recognition (Figure 5C). Syc-10 and Syc-59 are peptidomimetic inhibitors designed based on the Norwalk substrate [78] and synthesized by adding a protective bezyloxycarbonyl (CBZ) cap to the P3 residue and an aldehyde warhead to the P1 residue [82]. The carbon atom of the aldehyde warhead forms a covalent bond with the sulfur atom of the nucleophilic Cys139 residue. The P1 sidechain is inserted into the S1 pocket as expected, and forms hydrogen bonds with sidechains of His157 and Thr134 and makes van der Waals contacts with backbones of Ala159 and Ala160 in the S1 pocket. The hydrophobic P2 residue is accommodated by the S2 substrate-binding pocket, and its backbone amide interacts with Gln110 through a hydrogen bond. The P3 residue does not make any substantial contact with the viral protease. The N-terminal CBZ cap is involved in the van der Waals contacts with Ala160, Thr161, Vla168, Met107, and Ile109 residues located in the S4 pocket. The binding of these two inhibitors does not induce the flipping of the P136-G137 peptide as in the case of substrate binding. The extensive hydrogen bonds in the S1 and S2 pockets and the tight fit of the cap in the S4 pocket through hydrophobic interactions render these inhibitors very potent.

The successful synthesis and structural studies of the linear peptidomimetic inhibitors laid the foundation for the strategy of designing the current inhibitors by linking various warheads with the chemical backbone that mimics the protease cleavage sites. Replacement of P1 with lactam and cyanoacrylamide [83] and P2 with cyclohexylalanine [84] increased the inhibitor potency to the nanomolar range IC_50_ value. Exploration of the replacement of P1 residue with (S)-γ-lactam (compound **19**) or cyanoacrylamide (compound **43**) was enlightened by the previous substitution of acyclic amide to the P1 position, as the aldehyde warhead was highly reactive, which could lead to undesired off-target effects. The compounds exhibited sub-micromolar IC_50_ potency and micromolar EC_50_ potency when tested in the HG23 (Huh-7-based Norwalk virus replicon-bearing cell) system. Virtual docking of the compounds indicated that the differential positioning of the benzyl N-terminal cap may contribute to the different potencies. HuNoV protease showed a strong preference for a P2 cyclohexylamine in previous studies. The incorporation of this functional group into the phenyl ring stabilized the flexible benzyl cap by promoting hydrogen-bond formation to the Thr161 and Lys162 residues, as suggested by the x-ray crystallographic study. The cyclohexylalanine sidechain optimally filled the hydrophobic pocket of the S2 binding site, therefore improving the inhibitor activity to nanomolar IC_50_ and ED_50_ (compound **16**). Replacement of the aldehyde warhead with α-ketoamide, α-ketoheterocycle, or α-hydroxyphosphonate warheads led to other series of peptidomimetic inhibitors exhibiting anti-HuNoV activity in a cell-based replicon system [85,86]. The incorporation of α-hydroxyphosphonate was proven successful in the design of human renin inhibitors [87]. Compound **7**d possessing an α-hydroxy phosphonates warhead showed high enzyme selectivity, as it exhibited minimal inhibition to host proteases. However, in general, dipeptidyl inhibitors with aldehyde warheads have lower EC_50_ values compared to those of dipeptidyl inhibitors with α-hydroxyphosphonate or α-ketoamide warheads.

### 3.2. Prodrug

Certain linear compounds exhibited decreased inhibition potency when tested in cell-culture systems due to problems such as poor membrane permeability. A prodrug design can improve the solubility of the compounds and enhance the delivery of the compounds to the target location. The addition of a bisulfite adduct to the inhibitor warhead converted the transition-state HuNoV inhibitor into a prodrug that possessed potent pharmacological activity such as oral bioavailability and favorable absorption, distribution, metabolism, elimination, and toxicity (ADMET) characteristics to function as a latent-state form of the transition-state inhibitor, which is transformed into its active form when exposed to the gastrointestinal tract and blood plasma [88,89]. Bisulfite adducts have high aqueous solubility, and the transition between the precursor and the adduct compound is pH-dependent. These adduct compounds exhibited features that are important to bioavailability. A total of 24% of a bisulfite adduct compounds were metabolized by human liver microsomes after 1 h, indicating liver stability. The compound was bound strongly to human serum, suggesting potential good drug distribution [89]. The prodrugs with an aldehyde warhead, an isobutyl group, or a cyclohexylalanine group at the P2 position; a benzyl or a m-fluorobenzyl; or a 2-cyclohexylethyl cap exhibited in vitro inhibitory activities with micromolar inhibition constant. These prodrugs were then tested in an HG23 system, and the prodrug with an aldehyde warhead and a cyclohexylalalanine P2 group was the most potent [88]. The physicochemical and pharmacokinetics of the prodrugs could be further optimized by modification of the hydroxyl group in bisulfite adducts through ester and carbamate derivatization. The ester prodrugs with increased lipophilicity and the carbamate prodrugs derived from natural and unnatural amino acids exhibited enhanced absorption and better control of the hydrolysis. These prodrugs exhibited enhanced inhibitory activities when tested in the HG23 system. [89]. GC376, a dipeptidyl aldehyde bisulfite adduct prodrug, was that highly active against not only HuNoVs but also picornaviruses and coronaviruses which contain a conserved chymotrypsin-like fold and a catalytic triad with a nucleophile Cys [90,91]. The inhibitor exhibited sub-micromolar IC_50_ and EC_50_ inhibition potency when tested in the HG23 system. The binding of GC376, again, was mediated by the hydrogen bonds between the inhibitor and His30, Gln110, Thr134, His157, Ala158, and Ala160.

### 3.3. Macrocyclic Inhibitors

The structural studies with the linear peptidyl inhibitors indicated that HuNoV protease recognizes its ligand through a hydrogen-bonding network in the β-strand conformation. Macrocyclization, by tethering the first and last P residues of the inhibitors by a linker function group, preorganized the previous linear peptidyl inhibitor into a cyclic β-strand conformation to promote active site binding through the reduction of the entropy loss upon inhibitor binding [92]. As an effective method of depeptidization, macrocyclization has the advantage of mitigating the shortcomings of peptide-derived inhibitors, including proteolytic degradation, high conformational flexibility, and poor membrane permeability. HuNoV macrocyclic inhibitors are generally synthesized by tethering the P1 Gln residue and P3 residue of the linear inhibitor, as the P3 residue of the protease substrate exhibits a high level of flexibility, to linkers, usually with a size of 3 backbone carbons to ensure the formation of a β-strand conformation. The first HuNoV macrocyclic inhibitor was synthesized based on a previously reported aldehyde inhibitor and it exhibited moderate inhibitory activities to enterovirus, SARS-CoV, and HuNoV proteases [93]. Later, tethering with a 1,3,4-oxadiazole linker yielded a series of oxadiazole-based inhibitors including eight different compounds [94]. Inhibitory activity comparisons of these eight inhibitors indicated that the ring size is of paramount importance, as this feature determines the formation of an optimal β-strand conformation that allows for the proper docking of the inhibitors to the active site to correctly mediate the hydrogen bonds of the P1 and P2 residues with the protease. These oxadiazole-based inhibitors, the ones that exhibit both in vitro and in vivo inhibitory activities, had a ring size ranging from 16 to 20. Inhibitors with large ring sizes formed an extra hydrogen bond with Thr134, therefore exhibiting better in vitro inhibitory activities compared to inhibitors with short ring sizes. However, the short inhibitors did have a better cell permeability, whereas the inhibitor with a ring size of 21 exhibited poor cell permeability, possibly due to the absence of intramolecular hydrogen bonds, as revealed by the structural study, when tested in replicon cells. Noticeably, the substitution of a cyclohexylalanine at the P2 position did not result in significant improvement in the inhibitory activities, unlike in the case of linear inhibitors. Using a different linker, the researchers then synthesized another 11 triazole-based inhibitors [95]. These inhibitors, exhibiting decent in vitro inhibitory activities, had ring sizes ranging from 17 to 21 and had either an isobutyl, a cyclohexyl, or a phenyl cap. Again, the structural study indicated that the ring size affected the inhibitory activity by influencing the hydrogen bonding of the inhibitors to Thr134, His157, and the flexible backbone of the Thr123-Gly133 loop. Variations of the warhead and the P2 residue also had a significant impact on the potency of the macrocyclic inhibitors when tested both in vitro and in replicon cells [96]. In general, inhibitors with the aldehyde warhead and Leu or a cyclohexylalanine at the P2 position exhibited decent inhibitory activities. The binding of the macrocyclic inhibitors was facilitated by the hydrogen bonds that also existed in the substrate binding, including the ones between the inhibitor backbone and Gln110, Ala158, Ala160, the ones between the P1 glutamic acid sidechain and Thr134, and His157 [97] (Figure 5D).

### 3.4. Commercially Available Inhibitors

In addition to the synthesis of substrate-based inhibitors, several commercially available protease inhibitors have also been tested against HuNoV proteases. Serine protease inhibitor chymostatin was the first one to be tested and was found to inhibit Norwalk protease with an IC_50_ of around 10 µM [98]. More recently, some potent inhibitors for other proteases have also been tested. Deubiquitinase inhibitor WP1130 (also known as Desgrasyn) and its derivatives reduced Norwalk HuNoV replication when tested in an HG23 system [99]. WP1130 targeted the host deubiquitinase USP14 to activate the unfolded protein response [100]. The mechanism by which the unfolded protein response could inhibit HuNoV replication remains unclear. Rupintrivir, initially developed as an antiviral to treat rhinovirus infections, inhibited Norwalk protease and reduces Norwalk virus replication when tested in the HG23 system [101]. However, rupintrivir-resistant replicon cells were identified, and they had the Ala105 to Val and Ile109 to Val mutations [102]. This observation indicates that the generation of drug-resistant mutants can rapidly occur and inhibitors targeting different protease structural regions are needed.

## 4. Perspectives and Future Directions

**Figure 9:**
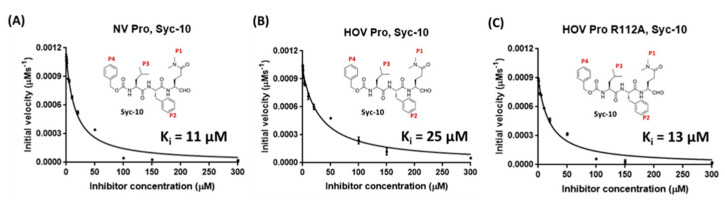
Peptidomimetic inhibitor exhibits differential inhibitory activity to proteases of different genogroups. (**A**) The inhibition potency of Syc-10 to GI.1. Syc-10 was identified and synthesized specifically to GI.1 protease. (**B**) The inhibition potency of Syc-10 to GII.4 protease is one-fold lower. (**C**) The inhibition potency of Syc-10 to a GII.4 Arg112 mutant.

The current strategy for developing HuNoV protease inhibitors is by targeting the active site and designing the inhibitors that bind to the substrate-binding pockets located near the active site. However, structural variations within the active site and substrate-binding pockets across different genogroups hamper the development of broad-spectrum inhibitors. As an example, a peptidomimetic inhibitor, Syc-10, exhibits potent inhibition to Norwalk protease but with a one-fold decrease in potency when targeting GII.4 Houston protease (Figure 9A,B). This is due to a conformational rearrangement of Arg112 sidechain induced by a flexible loop in the GII.4 protease, as discussed above. The inhibition potency was brought back only when the Arg112 was mutated into an Ala residue (Figure 9C). A molecular evolution study of the protease region of GII HuNoV indicated that it diverged from other HuNoV protease regions rapidly and evolved under no positive selection with a mutation rate of >10^−3^ substitutions/site/year [103]. Hence, it is necessary to identify regions that are highly conserved across the different genogroups and are essential for protease activity as potential binding sites for the antivirals. Recently, a study using crystal-based fragment screening has identified 15 inhibitors targeting regions besides the active site in Southampton protease [104]. The authors identified multiple fragment-based inhibitors to the regions, including the active site, the RNA-binding site, and the central cavity in the crystallographic tetramer interface. Interestingly, the binding of RNA to the protease, possibly adjacent to the active site, inhibited the proteolytic activities of both GI and GII proteases in vitro [105]. Therefore, identifying new lead compounds that target regions besides the active site could be a novel strategy for developing broad-spectrum HuNoV protease inhibitors.

### 4.1. Screening Approach—Lead Identification

A general strategy for identifying lead compounds is by screening compound libraries both chemically and virtually. High-throughput screening of chemical compound libraries to identify small molecules is an established technology. Typical libraries include the drug-like library, the natural product library, and the fragment library. Previous FDA-approved antivirals perhaps should also be considered as another library to be screened for HuNoV protease of the GII genogroup. In conjunction, virtual screening for the inhibitors can be performed using the available high-resolution protease structures. There is a large variety of compound databases available publicly, including, but not limited to, the MDDR library (180,000 compounds), the Available Chemical Directory (4 million compounds), and the ZINC (230 million compounds). A virtual screening starts by considering the druggability and structure flexibility of the target followed by choosing appropriate filters to remove undesirable compounds. Then, docking protocols with different levels of sophistication, including AutoDock, DOCK, FlexX, GOLD, and ICM, are applied to orient the ligands in the most favorable configuration within the target. At the end of the docking protocols, different post-filters are applied to inspect the evaluations and to limit the number of hits. Despite limitations such as the identification of false-positive compounds, high-throughput and virtual screening methods are likely to provide suitable leads for further optimization.

### 4.2. Function Group Modification—Lead Optimization

The optimization process aims to refine the identified compounds to generate more potent leads that possess absorption, distribution, metabolism, and excretion (ADME) properties, as well as physicochemical and pharmacokinetic (PK) properties adequate to be examined in an in vivo system. These properties are essential to evaluate the efficacy and safety of the lead compounds. The majority of optimization efforts are devoted to improving compound efficacy. The optimization process involves extensive modifications of the functional groups around the core compound structure and measurements of activities of the modified compounds in a systematic manner. The compounds can be modified by alteration of the ring systems, derivatization of the functional groups, and isosteric replacement. A batch of analogs is usually synthesized at this stage. After initial optimization, the binding affinities of the compounds are improved to the nanomolar range. This involves extensive structure–activity relationship (SAR) investigations that require structure details of the target to modify the hit compounds. After improving the compound efficacy, the ADMET profile is optimized. To improve the solubility, the most straightforward approach is to incorporate hydrophilic functional groups into the compounds. Poor cell membrane permeability hinders the delivery of the compounds to the target in the cells, resulting in decreased in vivo potency. To improve cell membrane permeability, the overall lipophilicity of the compounds has to be modified by either adding lipophilic fragments or selectively removing hydrophilic fragments. Toxicity caused because of off-target effects can be addressed by a structural modification to improve the specificity of the compounds. A prodrug is an approach frequently used to improve oral bioavailability, which can also reduce toxicity and improve metabolic stability. Therefore, the prodrug approach is an important strategy to be considered when developing lead compounds into HuNoV therapeutic antivirals. Compounds that can maintain favorable inhibitory activities after optimization are ready to be tested in vivo.

### 4.3. In Vivo Testing—Lead Validation

The examination of screened inhibitors has long been hampered by a lack of a robust in vitro HuNoV culture system for decades. Therefore, most synthesized inhibitors were tested against HuNoV using a replicon system. Later, as a cell-based approach, murine HuNoVs that replicate in murine macrophage cell lines [106] have been used as in vitro culture systems to test the antiviral effects of the inhibitors. More recently, breakthroughs have been made in zebrafish larvae, B-cell, and human intestinal enteroid (HIE) culture systems to support HuNoV replication [107,108,109]. For example, a protocol has recently been developed to evaluate antiviral effect and toxicity of small molecules in HuNoV-infected zebrafish larvae [110]. Using this model, a novel peptidomimetic protease inhibitor, which is structurally related to rupintrivir, has been shown to exhibit potent inhibition of HuNoV [111]. The primary site of infection for HuNoV is the enterocyte from the small intestine (duodenum, jejunum, and ileum) [112]. Given the evidence that HuNoV can replicate in B cell-deficient patients [113,114], and that the noroviral antigen is not detected in B cells in the lamina propria [112], testing of the inhibitors in HIEs, which more closely resembles the HuNoV infection site, is likely more physiologically suitable. The replication cycle and pathogenesis of HuNoV have been studied in many large-animal models, including pig, calves, rhesus macaque, and chimpanzees [115]. Although these animal models contribute significantly to the understanding of HuNoV biology, they are not ideal for large-scale antiviral testing experiments. A murine mouse model with a BALB/c background deficient in recombination activation gene (Rag) common gamma chain (Rag^−/−^γc^−/−^) has been reported to support replication of GII.4 HuNoV [116]. However, this mouse model only supports a limited replication of HuNoV, and inoculation via the oral route is not sufficient to cause disease. Mice infected with HuNoV do not develop clinical symptoms, which further complicates the ability to assess the efficacy of potential inhibitors. Instead of oral administration, intraperitoneal injection of small compounds is used as an alternative method to avoid potential delivery problems. The mice may also respond to antiviral treatments in ways that differ from humans given the genetic and physiological differences between the species. A humanized mouse model might be an ideal alternative if such a model can be developed. It is essential to evaluate the efficacy and toxicity of the inhibitors in vivo before proceeding to clinical characterizations.

### 4.4. Conclusions

HuNoV infections are a significant worldwide health burden for all age groups, but particularly for the young, aged, and immunocompromised populations. No approved antiviral therapeutics are currently available for HuNoV infection. Robust post-infection therapeutics would be beneficial for these populations. HuNoV protease is a prime drug target given its importance in mediating viral replication. Recent studies have identified a large variety of substrate-based lead compounds that effectively inhibit HuNoV protease of certain genotypes by targeting the active site. However, the structural differences within the active site and the substrate-binding pockets hamper the development of broad-spectrum inhibitors. A comparative analysis of the available HuNoV protease structures indicates that other regions besides the active site can be targets for developing novel broad-spectrum HuNoV protease inhibitors. It is hoped that further development of protocols for optimization of such inhibitors along with robust methods for in vivo assessment will lead to effective antivirals to counter global HuNoV infections.

## Data Availability

Not applicable.
